# Blunt Trauma and Right Diaphragmatic Rupture: Unveiling the Impact

**DOI:** 10.7759/cureus.40788

**Published:** 2023-06-22

**Authors:** Duarte Gil Alves, Jessica Sousa, João Eurico Reis, Rómulo Ribeiro, Paulo Calvinho

**Affiliations:** 1 General Surgery, Hospital Dr. Nélio Mendonça, Funchal, PRT; 2 Radiology, Centro Hospitalar Universitario do Porto EPE, Porto, PRT; 3 Thoracic Surgery, Hospital Santa Marta, Lisboa, PRT

**Keywords:** hemopneumothorax, osteosynthesis, surgical fixation devices, high energy trauma, blunt chest injuries, blunt thoracic trauma, explorative laparotomy, emergency thoracotomy, traumatic diaphragmatic rupture, diaphragmatic rupture

## Abstract

Traumatic diaphragmatic ruptures are rare, yet blunt injuries tend to be more easily overlooked compared to penetrating trauma. The minimal evidence of external injuries makes a high index of suspicion key for diagnosis. We report the case of a right-sided thoracoabdominal blunt trauma that resulted in a diaphragmatic rupture and fractured rib. Although often approached through a midline laparotomy, a definitive right thoracotomy repair was exceptionally performed since the adjacent peritoneum remained uninjured.

## Introduction

Blunt traumatic diaphragmatic ruptures have a low incidence ranging from 1% to 7%, yet they are often associated with serious injury or death owing to a high-energy traumatic cause [1,2]. Although the most common mechanism of injury is road traffic collisions, any event that triggers an increased intra-abdominal pressure is likely to cause a diaphragm rupture [3].

The left hemidiaphragm is two to three times more prone to injury than the right [4,5]. Besides a probable congenital weakness of the left leaflet, the liver may have a protective role in attenuating the high-energy transfer [4]. However, some authors believe that the proportion of left- and right-sided diaphragmatic ruptures would be balanced if we were to evaluate their prevalence during autopsies. Given that the right diaphragm ruptures are associated with liver injuries as opposed to spleen lacerations on the left, they have a higher mortality and therefore tend to be more commonly undiagnosed [6].

While most rib fractures can be successfully managed through a conservative approach, surgical rib fracture fixation might be an advantageous treatment option in selected patients [7]. Since a surgical approach is effective in enhancing pulmonary function and reducing trauma-associated pain, patients with rib fractures associated with impending respiratory failure or flail chest are generally accepted as the most suitable surgical candidates [8,9].

Considering that there are no pathognomonic findings suggestive of blunt traumatic diaphragmatic ruptures, emergency physicians should keep a high index of suspicion, even more so if there is evidence of other associated high-energy injuries.

## Case presentation

A 22-year-old male with no relevant medical history suffered right-sided thoracoabdominal blunt trauma after hitting a rail during a wakeboarding competition. Prehospital emergency care services provided prompt stabilization and transported the individual to a level III trauma center.

Upon arrival to the emergency department, a primary survey was performed according to the European Trauma Course (ETC) protocols. The patient’s airway was patent with a respiratory rate of 24 cycles per minute and a peripheral oxygen saturation of 91% on a 5L/min oxygen flow rate delivered by face mask with reservoir. The chest examination revealed decreased respiratory sounds on the right, subcutaneous emphysema, and crepitus following posterior palpation of the right ribs. Regarding circulation, the patient had a blood pressure of 82/58 mmHg, a heart rate of 112 beats per minute, and no external signs of active bleeding. The patient was conscious and oriented.

Arterial blood gas (ABG) analysis revealed a pH of 7.39, a partial pressure of carbon dioxide (PaCO_2_) of 36 mmHg, a partial pressure of oxygen (PaO_2_) of 60 mmHg, oxygen saturation of 90% (SaO_2_), bicarbonate (HCO_3_) of 21.8 mEq/L, hemoglobin of 13.1 g/dL, hematocrit of 41%, and lactates of 1.7 mg/dL. After two peripheral 16G IV lines were inserted, the patient was given 1 gram of tranexamic acid and was started warmed Ringer’s lactate fluid resuscitation.

**Figure 1:**
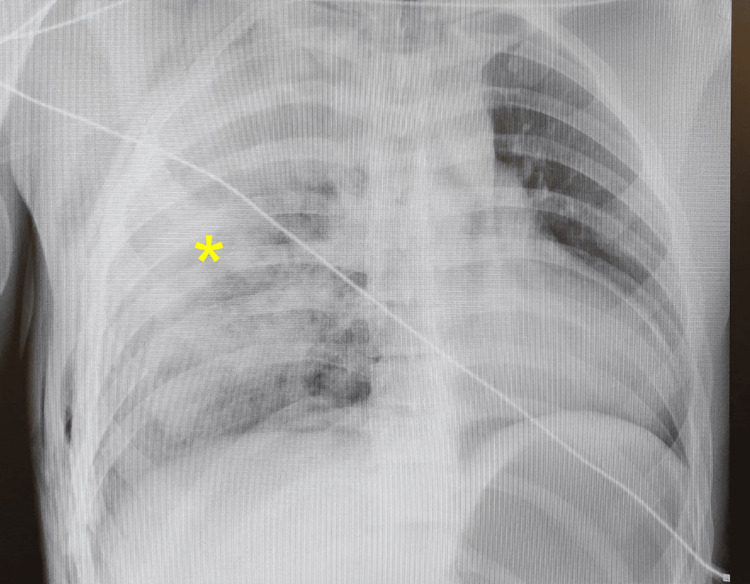
Chest X-ray revealing a right unilateral whiteout and subcutaneous emphysema involving the right thoracic wall resulting from a right hemopneumothorax (yellow asterisk).

**Figure 2:**
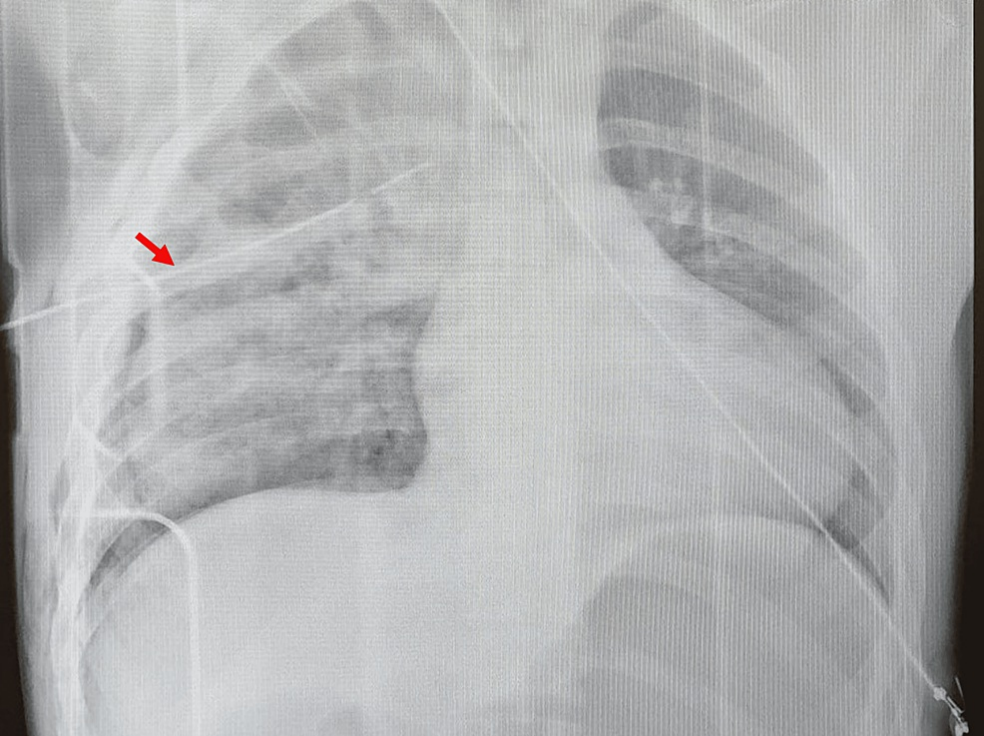
Chest X-ray after a right hemithorax decompression with a chest tube placed in the fourth intercostal space (red arrow).

A plain chest X-ray was performed, which revealed the presence of a right hemopneumothorax (Figure 1). Given that the patient was hypoxemic, tachycardic, and hypotensive, surgeons decided to decompress the right hemithorax by placing a chest tube in the right fourth intercostal space (Figure 2). The immediate drainage of 1,300 mL of blood from the thoracic cavity was observed. Since the patient’s vital signs improved and the patient became hemodynamically stable, a computed tomography (CT) scan of the thorax, abdomen, and pelvis was performed.

**Figure 3:**
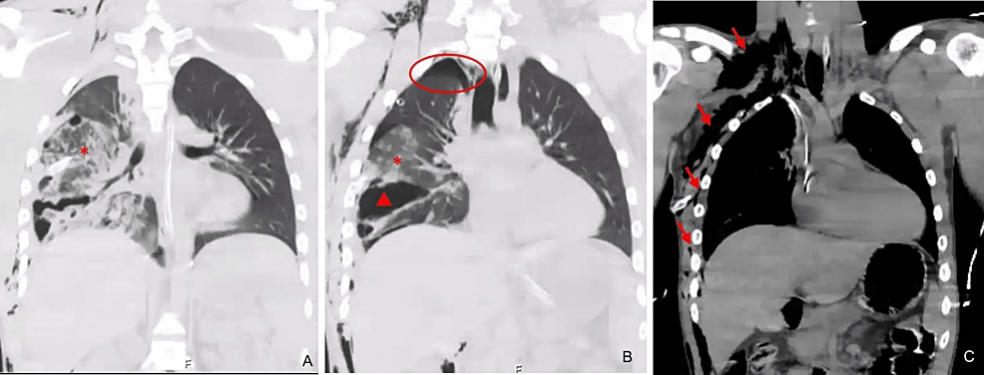
Coronal CT reconstructions from the lung (A, B) and mediastinal (C) windows revealing a right pneumothorax (circle), extensive pulmonary contusion (*), and laceration (triangle) of the right lung parenchyma. It also shows an extensive subcutaneous emphysema involving the right thoracic wall that extends to the neck (arrows).

**Figure 4:**
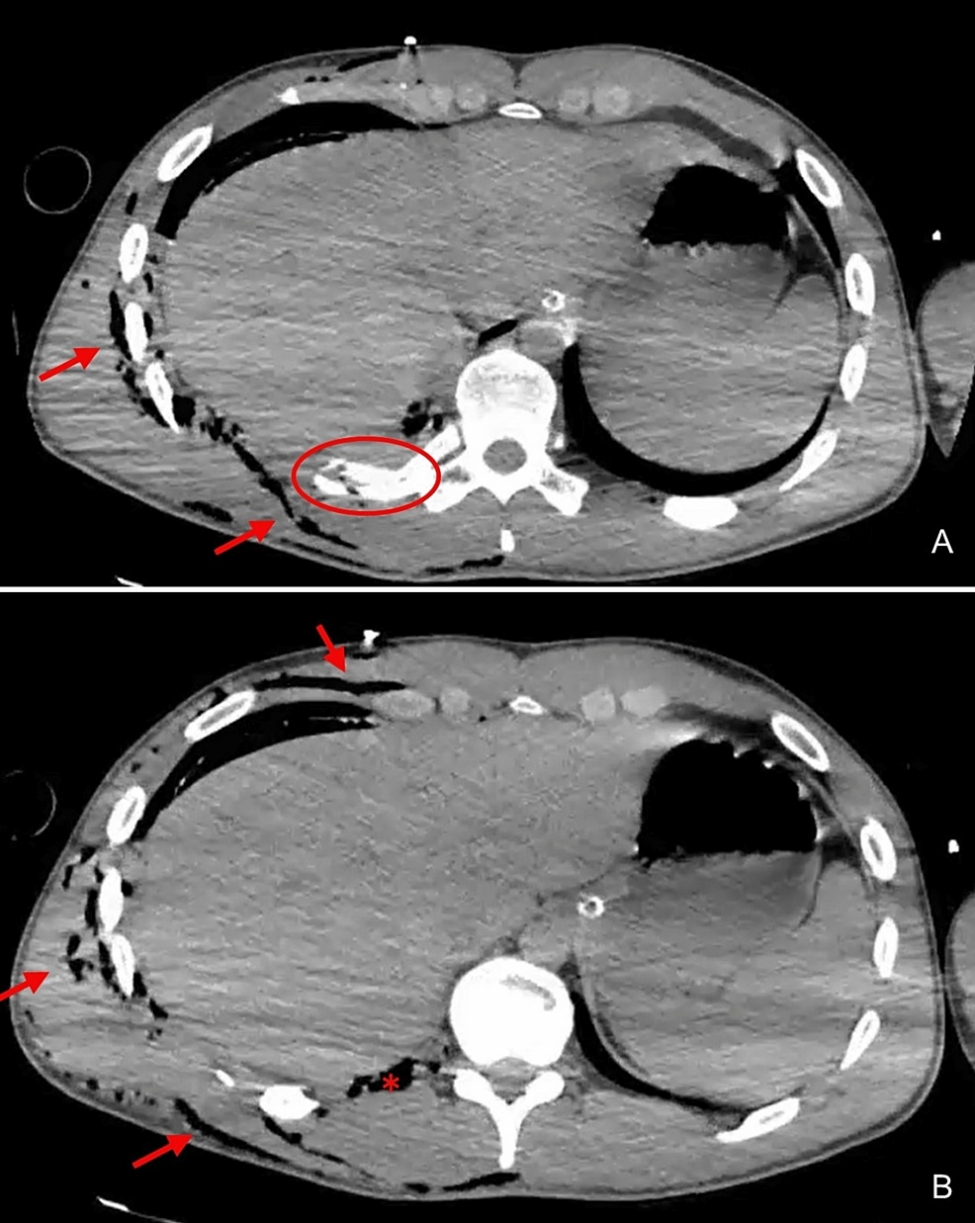
Axial CT acquisitions showing a posterior misaligned fracture of the right 10th rib (circle), extensive subcutaneous emphysema involving the right thoracic wall and extending to the neck and ipsilateral abdominal wall (arrows), and area of detachment of the diaphragm from the posterior thoracic wall (*) next to the fractures rib.

**Figure 5:**
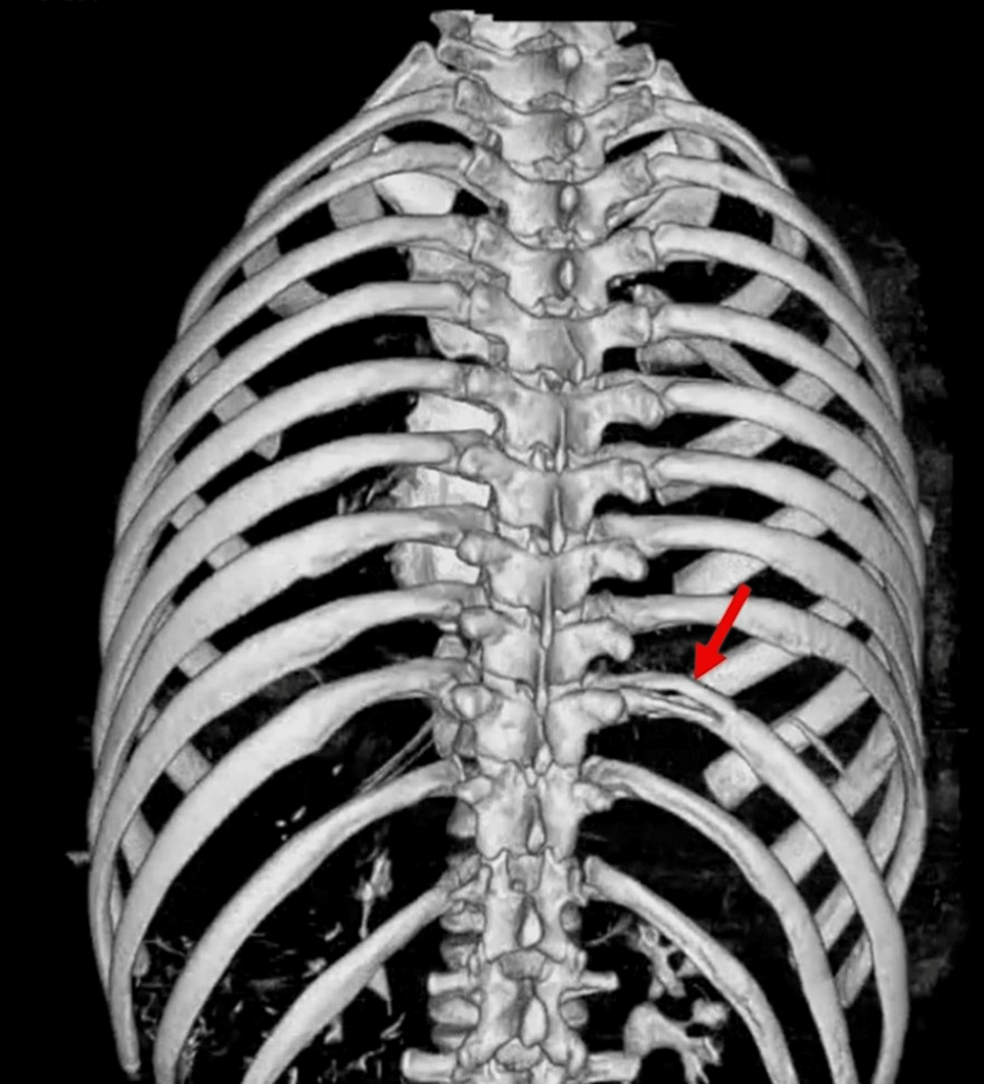
Three-dimensional CT reconstruction showing a posterior misaligned fracture of the right 10th rib (arrow).

The CT scan with contrast revealed a right pneumothorax of inferior predominance and a moderate hemothorax with the chest tube positioned in the right lung fissure. An extensive right pulmonary contusion sparing only the upper lobe and a misaligned fracture of the 10th rib were also identified. The CT scan confirmed subcutaneous emphysema on the right involving the right thoracic wall and extending to the neck and ipsilateral abdominal wall (Figures 3A-3C). An aligned fracture of the spinous process of D7 was also diagnosed. In the abdomen, there was a thin layer of supramesocolic pneumoperitoneum on the right side that appeared to communicate with the subcutaneous emphysema at the level of the diaphragmatic dome, suggesting a probable minor local detachment (Figures 4A-4C, Figure 5).

 

After undergoing the CT scan, an additional 300 mL of blood drained from the chest tube, and the patient experienced a recurrence of decreased blood pressure. A full two units of crossmatched packed red blood cells were requested and administered. Since the patient experienced ongoing bleeding from the thoracic cavity and there was no surgical expertise available on site for an urgent surgical review, the decision was made to transfer the patient by medical helicopter to the closest level I trauma center, which was located 200 km away.

Upon arrival, the patient underwent an exploratory laparotomy that found neither intra-abdominal organ injuries nor lacerations or tears to the diaphragmatic dome. At the same operating time, an exploratory thoracoscopy was performed to inspect the right-sided diaphragm. During the procedure, aside from the pulmonary contusions, surgeons confirmed a detachment of the diaphragm from the posterior thoracic wall with active oozing originating from the ruptured muscle (Video 1). No communication between the thorax and abdomen was identified, owing to the integrity of the peritoneum.

**Video 1:** Exploratory thoracoscopy showing the posterior right-sided diaphragmatic rupture with some active oozing from the diaphragm laceration.

**Figure 6:**
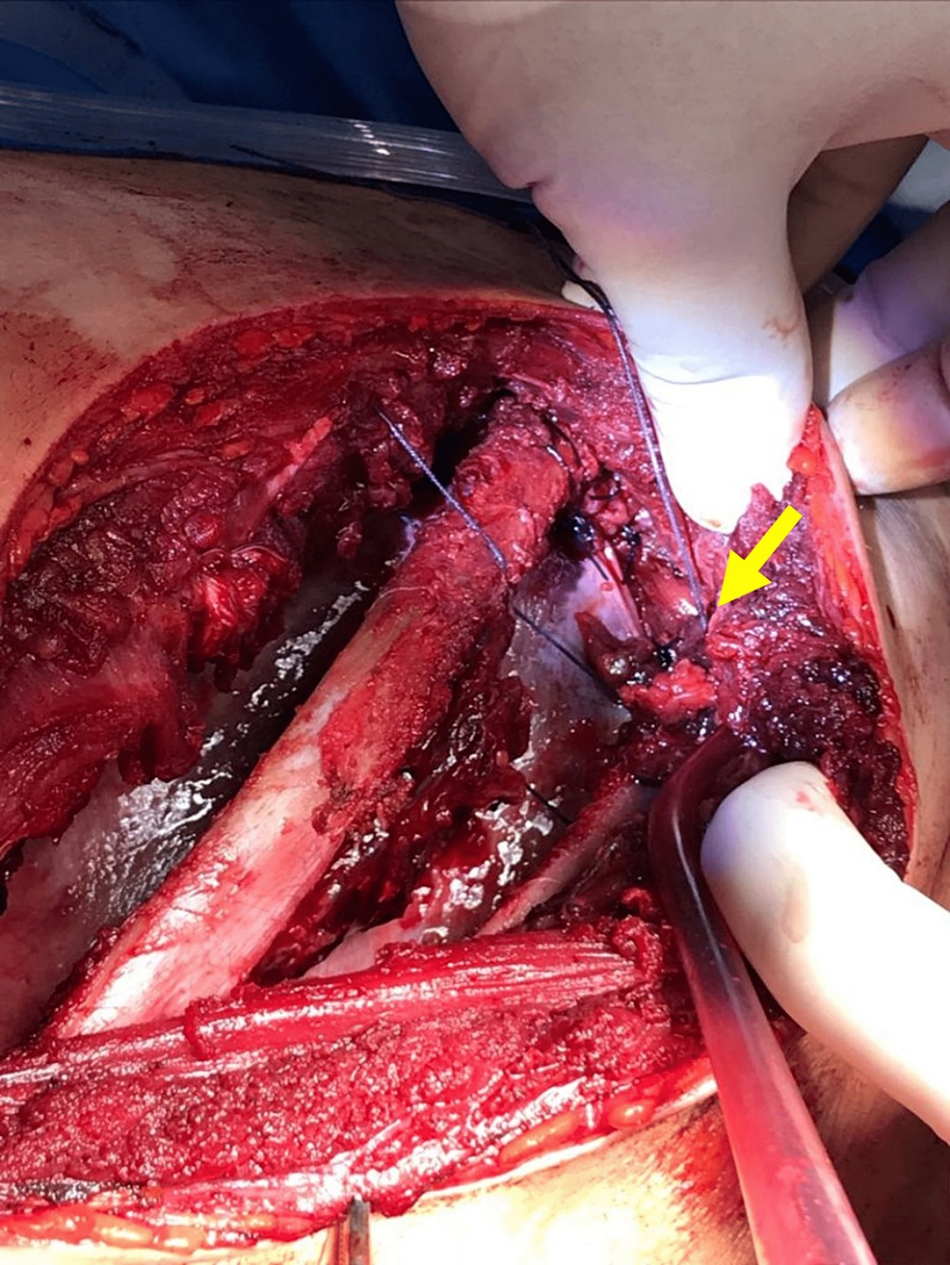
Repair of the diaphragmatic defect by non-absorbable interrupted suturing using polypropylene (yellow arrow).

**Figure 7:**
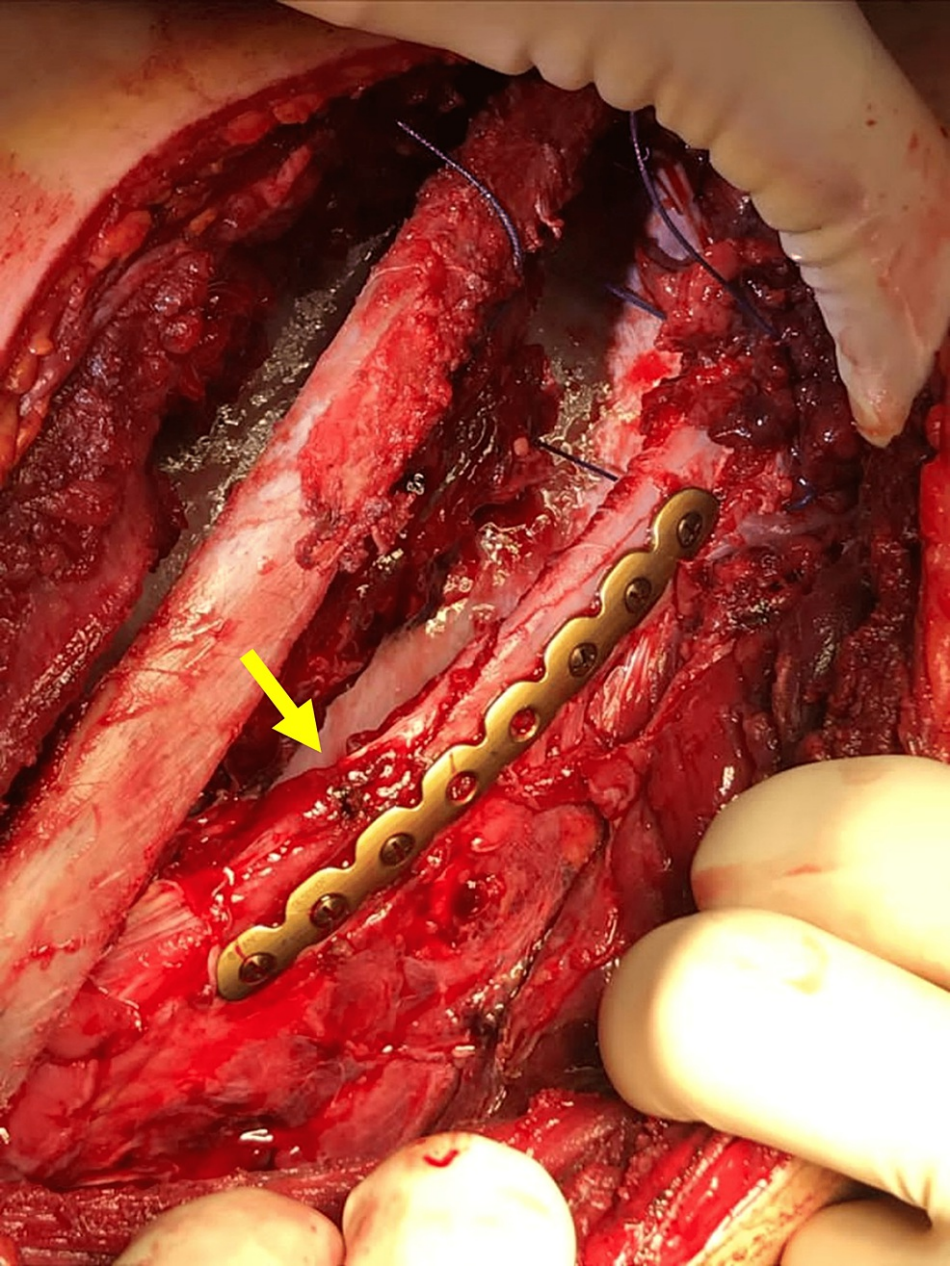
Osteosynthesis of the eight fractured rib using the MatrixRIB® system (yellow arrow).

**Figure 8:**
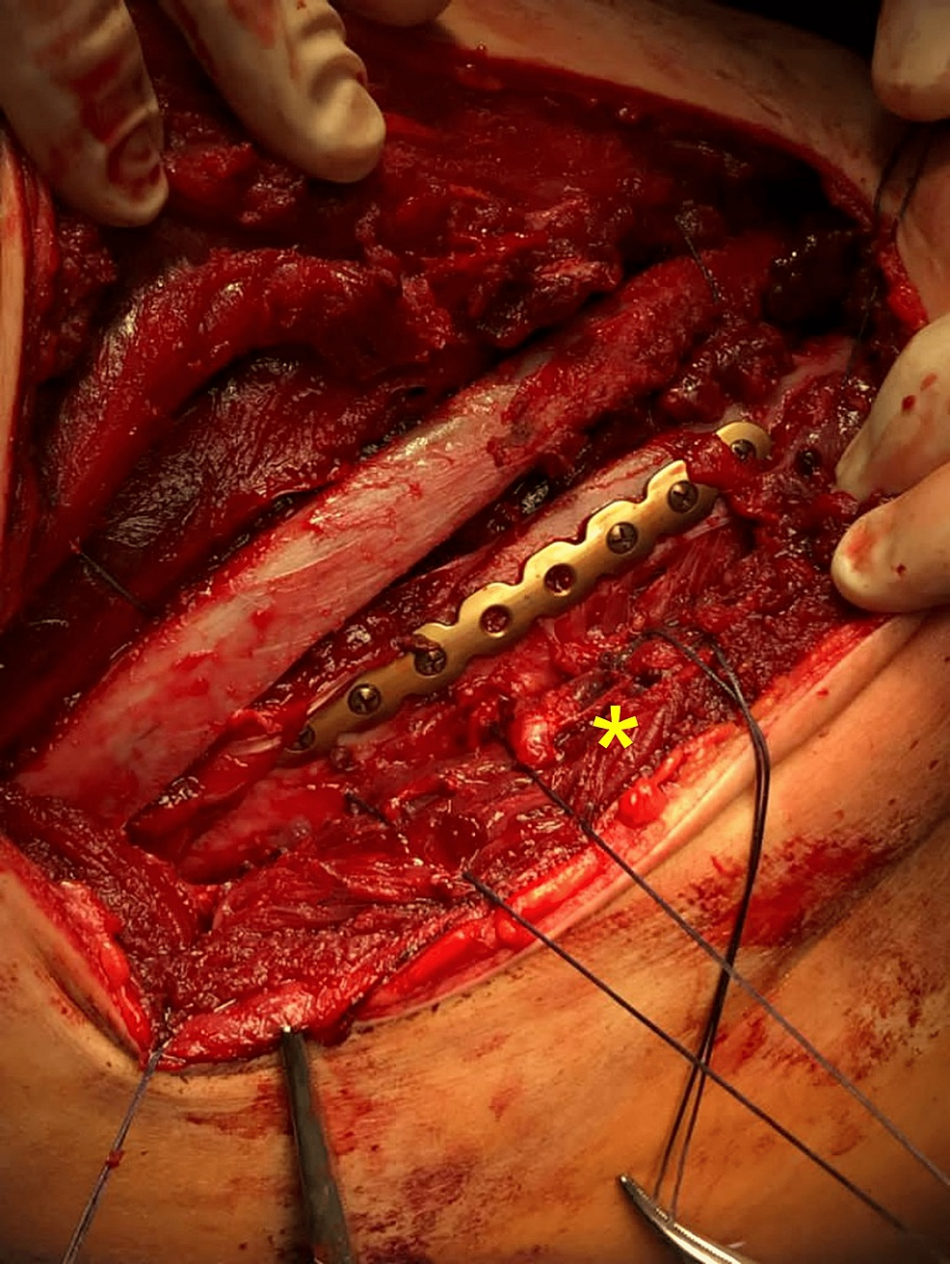
Final surgical view before the closure of the intercostal muscles layer (yellow asterisk).

**Figure 9:**
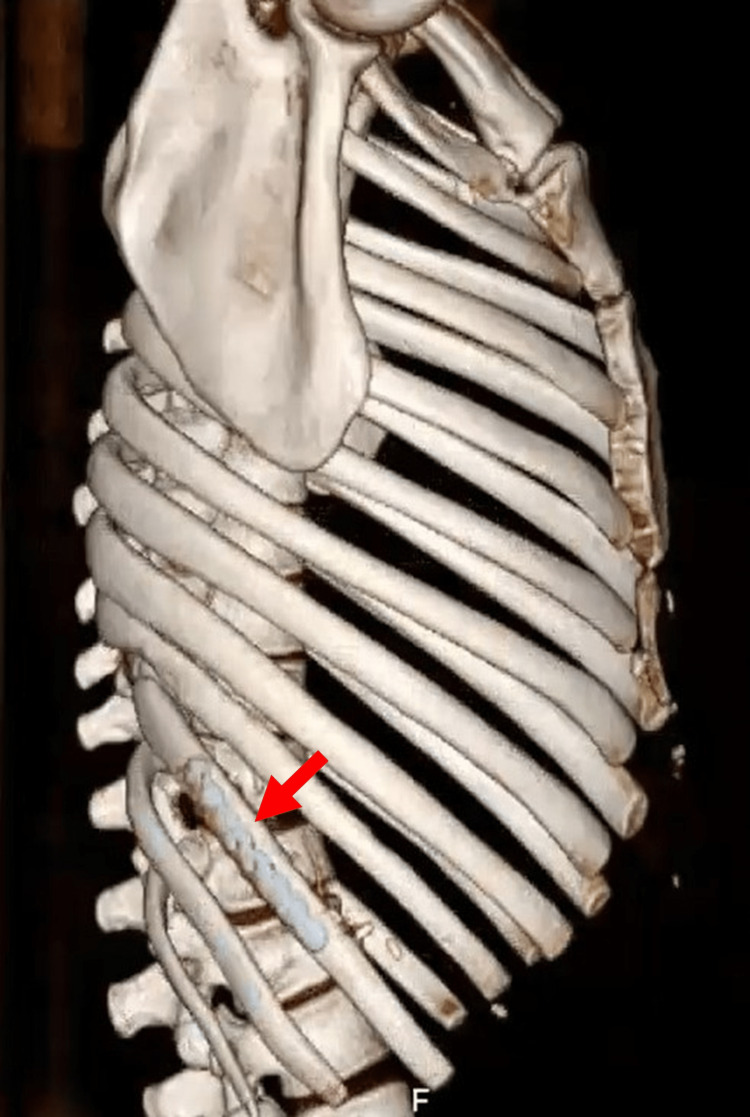
Three-dimensional CT reconstruction showing the osteosynthesis of the 10th fractured rib using the MatrixRIB® system (red arrow).

After converting to open thoracotomy at the eight intercostal space and achieving hemostasis of the ruptured diaphragm muscle, surgeons repaired the diaphragmatic defect with a non-absorbable interrupted sutures using polypropylene (Figure 6). Osteosynthesis of the 10th fractured rib using the MatrixRIB® system was also performed. Surgeons were able to achieve proper rib fixation using a pre-contoured 1.5-mm plate with eight holes and a minimum of three self-tapping locking 12-mm screws on each side of the fracture (Figures 7-9). Two 28 Fr chest tubes were left in the apical and basal right hemithorax.

 

The patient was then taken to an intensive care unit, where he spent the first 48 hours before being downgraded to a medical-surgical unit afterward. On postoperative day 4, the two 28 Fr tubes were safely removed, as drainage volumes had gradually diminished and the right lung had properly re-expanded.

After three months of follow-up, a CT scan of the thorax revealed no significant findings or sequelae regarding the diaphragmatic repair nor the rib osteosynthesis. The patient did not present symptoms of cardiorespiratory compromise or chronic pain associated with rib fixation.

## Discussion

In a thoracoabdominal blunt trauma, the minimal external evidence of injuries may pose a challenge during the physical examination. Therefore, it is of utmost importance to understand the mechanism of trauma, as a direct lateral impact to the thorax can result in the avulsion of the diaphragm insertions from the ribs [6].

The gold standard CT examination approach for hemodynamically stable trauma victims plays a major role in the diagnosis of diaphragm injuries owing to its high specificity and sensitivity [10]. However, if the results are negative or inconclusive, surgeons may use laparoscopy and/or thoracoscopy to rule out diagrammatic injury. Diagnostic thoracoscopy is even more relevant in the diagnosis of posterior right-sided diaphragmatic injuries [11].

Only half of the diagrammatic ruptures happen as isolated injuries, and only one-third of them are associated with rib fractures [12,13]. The identification of a rib fracture severe enough to cause misalignment should therefore prompt the surgeon to further investigate possible underlying trauma, specifically trauma involving the first three ribs or the lower ribs. While fractures of the first three ribs are associated with a high degree of energy transfer, fractures of the lower ribs carry a higher risk of liver and spleen injury. Since the primary injury tends to be easily missed and remains undiagnosed, complications arising from blunt traumatic diaphragmatic ruptures are much more common compared to penetrating trauma [14].

Rib fracture fixation using locking plates is an effective technique as it not only restores the chest wall’s anatomy and stability but also reduces pain and improves patients’ recovery [15]. Despite the absence of anticipated respiratory failure or chest wall instability related to the misaligned rib fracture in this case, as a way of minimizing postoperative morbidity, surgeons chose to perform an opportunistic rib osteosynthesis during the closure of the thoracotomy prompted by the diaphragmatic rupture.

A midline laparotomy is an often-recommended approach, as it allows for the examination of the abdominal surface of the diaphragm and repair if needed [16]. In the case described, a definitive surgical approach by thoracotomy was achieved by reattaching the right-sided diaphragm and stabilizing the rib fracture given the peritoneum’s integrity. By restoring the diaphragm anatomy, surgeons were able to avoid the risks of herniation, often presenting as obstruction, strangulation, and rupture in a delayed phase [17].

Since there was no peritoneum disruption, by initially submitting the patient to a thoracic approach, surgeons could have performed rib fixation, achieved hemostasis, and repaired the ruptured diaphragm muscle solely through thoracotomy. Thus, sparing the patient added surgical morbidity. However, confronted with the possible existence of pneumoperitoneum reported by the CT scan, the consequent risk of a missed abdominal injury, and considering their surgical experience, surgeons decided to proceed with an initial laparotomy approach.

## Conclusions

When faced with evidence of high-energy injuries, one must have a high clinical index of suspicion to properly diagnose and manage traumatic diaphragmatic ruptures. The risk of death directly related to diaphragmatic rupture alone is 25%, while the associated morbidity is significant, reaching up to 60%. Therefore, once identified, repair should be performed either through open or minimally invasive surgery. A thoracic and/or abdominal approach must be decided upon based on the presence of other intra-abdominal or thoracic injuries and the surgeon’s experience.
